# Evaluator-blinded trial evaluating nurse-led immunotherapy DEcision Coaching In persons with relapsing-remitting Multiple Sclerosis (DECIMS) and accompanying process evaluation: study protocol for a cluster randomised controlled trial

**DOI:** 10.1186/s13063-015-0611-7

**Published:** 2015-03-21

**Authors:** Anne Christin Rahn, Sascha Köpke, Jürgen Kasper, Eik Vettorazzi, Ingrid Mühlhauser, Christoph Heesen

**Affiliations:** Institute for Neuroimmunology and Clinical MS Research, University Medical Center Hamburg-Eppendorf, Martinistraße 52, D-20246 Hamburg, Germany; Unit of Health Sciences and Education, MIN Faculty, University of Hamburg, Martin-Luther-King-Platz 6, D-20146 Hamburg, Germany; Nursing Research Unit, Institute of Social Medicine and Epidemiology, University of Lübeck, Ratzeburger Allee 160, D-23538 Lübeck, Germany; Faculty of Health Sciences, Department of Health and Care Sciences, University of Tromsø, MH Building, N-9037 Tromsø, Norway; Department of Medical Biometry and Epidemiology, University Medical Center Hamburg-Eppendorf, Martinistraße 52, D-20246 Hamburg, Germany; MS Day Hospital and Outpatient Unit, Department of Neurology, University Medical Center Hamburg-Eppendorf, Martinistraße 52, D-20246 Hamburg, Germany

**Keywords:** Multiple sclerosis, Coaching, Shared decision-making, Cluster randomised controlled trial, Patient information, Nurses, Self-management, Evidence-based medicine

## Abstract

**Background:**

Multiple sclerosis is a chronic neurological condition usually starting in early adulthood and regularly leading to severe disability. Immunotherapy options are growing in number and complexity, while costs of treatments are high and adherence rates remain low. Therefore, treatment decision-making has become more complex for patients. Structured decision coaching, based on the principles of evidence-based patient information and shared decision-making, has the potential to facilitate participation of individuals in the decision-making process.

This cluster randomised controlled trial follows the assumption that decision coaching by trained nurses, using evidence-based patient information and preference elicitation, will facilitate informed choices and induce higher decision quality, as well as better decisional adherence.

**Methods/Design:**

The decision coaching programme will be evaluated through an evaluator-blinded superiority cluster randomised controlled trial, including 300 patients with suspected or definite relapsing-remitting multiple sclerosis, facing an immunotherapy decision. The clusters are 12 multiple sclerosis outpatient clinics in Germany. Further, the trial will be accompanied by a mixed-methods process evaluation and a cost-effectiveness study.

Nurses in the intervention group will be trained in shared decision-making, coaching, and evidence-based patient information principles. Patients who meet the inclusion criteria will receive decision coaching (intervention group) with up to three face-to-face coaching sessions with a trained nurse (decision coach) or counselling as usual (control group). Patients in both groups will be given access to an evidence-based online information tool.

The primary outcome is ‘informed choice’ after six months, assessed with the multi-dimensional measure of informed choice including the sub-dimensions risk knowledge (questionnaire), attitude concerning immunotherapy (questionnaire), and immunotherapy uptake (telephone survey). Secondary outcomes include decisional conflict, adherence to immunotherapy decisions, autonomy preference, planned behaviour, coping self-efficacy, and perceived involvement in coaching and decisional encounters. Safety outcomes are comprised of anxiety and depression and disease-specific quality of life.

**Discussion:**

This trial will assess the effectiveness of a new model of patient decision support concerning MS-immunotherapy options. The delegation of treatment information provision from physicians to trained nurses bears the potential to change current doctor-focused practice in Germany.

**Trial registration:**

Current Controlled Trials (identifier: ISRCTN37929939), May 27, 2014.

**Electronic supplementary material:**

The online version of this article (doi:10.1186/s13063-015-0611-7) contains supplementary material, which is available to authorized users.

## Background

Multiple sclerosis (MS) is a chronic, inflammatory, autoimmune disorder, which is characterised by destruction of myelin in the central nervous system. The disease affects mainly young adults, with an average age of onset of around 30 years [1,2].

Around 2,000,000 people worldwide are affected with MS and at least 120,000 people in Germany have MS [3]. Further, recent insurance company based numbers have estimated there to be around 180,000 affected people in Germany [4]. There are between 3,000 to 5,000 new cases every year in Germany (four to six per 100,000).

Due to the long course of this disease and resulting severe disabilities, MS is of major health economic relevance [5]. Annual costs per patient in Europe are estimated at €18,000 for mild MS (Expanded Disability Status Scale (EDSS) <4.0), €36,500 for moderate MS (EDSS 4.0 to 6.5) and €62,000 for severe MS (EDSS >7.0) [6]. Total societal costs in Germany have been estimated at around €4,000,000,000 in 2001 [3].

Due to many uncertainties such as the possibility of a benign variant of MS [7,8], and unclear long-term benefits of treatments, some of them with life-threatening risks [9], immunotherapy decisions are not straightforward. In addition, recent studies have shown non-adherence rates of up to 50% within the first two years of treatment [10]. Thus, immunotherapy decision-making and decisional adherence are of high personal and societal relevance.

A shared decision-making (SDM) approach is currently regarded as the ideal approach in medical decision-making, based on the ethical principle of patient autonomy and on patient preferences [11]. A prerequisite of SDM is the availability of balanced and understandable information emphasising the crucial position of evidence-based patient information (EBPI) in this process [11]. A second aspect of SDM is self-reflection on values and preferences, which might substantially differ between patients and physicians [12]. This ideal concept of informed SDM is confronted with the current situation of medical care in Germany and other European countries, characterised by an increased burden of work for increasingly fewer physicians [13].

During recent years, so-called MS specialist nurses have been established, partially with the support of pharmaceutical companies for coaching patients on injectable treatments [14]. Although in some countries nurses already have active roles [15], there has been no widespread, systematic integration of MS nurses into immunotherapy decision-making processes based on EBPI. Coaching, provided in a structured manner and according to the principles of EBPI, can facilitate participation of individuals in the decision-making process. In this trial the following coaching definition of Stacey *et al*. [16] is applied: ‘Coaching is defined as the provision of support by a trained individual (either in person or remotely *-* for example by telephone or internet), who is supportive but non-directive, for a patient or family facing a decision’ [16]. Further, decision coaching is determined by the inclusion of SDM and EBPI components, as for example the assessment of patients’ decision-making needs, provision of information on benefits and harms of each option, and the facilitation and monitoring of the decision-making progress [16].

In a recent systematic review [17], decision coaching provided along patient decision aids has been summarised based on trials reviewed in a Cochrane review [18]. The systematic review could not show a benefit regarding knowledge improvement compared to provision of patient decision aids only. For other outcomes, the trials produced diverse results, yet no negative effects have been demonstrated. Due to these findings and the limited number of trials, the authors concluded that further research in this area is needed [17]. However, in those trials where coaching has been provided by nurses, results are in general more promising [19-21].

We assume that beyond thoroughly developed decision support technologies and advanced communication concepts, structural change in clinical decision-making is essential for successful implementation of patient involvement into clinical practice. Therefore, this trial aims at clarifying the possible gains of, and also barriers to, giving MS nurses a crucial role in immunotherapy decision-making processes. The nurse decision coach model has been developed to redistribute health professionals’ tasks in supporting patients’ decision-making processes [22]. Here, the physician encounter is supplemented by the provision of an evidence-based online patient information tool (DECIMS-Wiki) and up to three decision coaching sessions with specialist MS nurses (decision coaches) supporting patients to process the information, to clarify patients’ own values, and to identify personal barriers in the decision-making process before a decision is made. By this stepwise structured and individualised process, we expect patients to be able to deeper elaborate their own decisions and to more actively participate in decision processes. Clarification of patients’ own values, identification of barriers, evidence-based information, and participation in decision processes are prerequisites for patients in order to make informed choices and to achieve high decision quality.

This protocol has been developed and structured following the recommendations of the Standard Protocol Items: Recommendations for Interventional Trials (SPIRIT) 2013 statement for clinical trial protocols [23]. Please see Additional file 1 for the complete SPIRIT checklist. Further, the Consolidated Standards of Reporting Trials (CONSORT) extensions for cluster randomised trials and for randomised controlled trials of non-pharmacologic treatments have been considered and will be used for reporting study results [24,25].

A recent Cochrane review showed that decision aids [26] in health treatment enhance accurate expectations and increase patient involvement. Also patient-physician communication is positively influenced if values are explicitly clarified. However, effects on decisional adherence and health outcomes remain inconsistent. In another Cochrane review [27] on interventions for health professionals to enhance SDM, all three trials out of 39 trials using a nurse-based educative intervention showed changes in consultations [28] and on patient relevant outcomes [29,30], stressing the relevance of this approach. In addition, our own Cochrane review on information provision interventions in MS identified 10 randomised controlled trials with heterogeneous approaches and inconsistent results [31].

Since 2001, we have studied EBPI and SDM in MS and conducted four controlled trials [32]. All interventions were based on the concept that more patient involvement through carefully developed information leads to a greater sense of control and empowers patients for disease-specific self-management especially regarding treatment decision making. While epidemiological studies in MS have consistently shown that objective and perceived stress is a relevant relapse risk factor [33], altered psychological factors might even impact on the overall disease process [34]. Our first randomised controlled trial clearly showed altered health behaviour in MS relapse management after a four-hour educational intervention in a cohort of 150 MS patients followed up on for two years [35]. Interestingly, trained patients had less relapses. On the other hand, a printed EBPI on immunotherapy alone was not sufficient to alter decision-making processes in another trial [36].

Other groups have engaged in the evaluation of patients’ attitudes and risk behaviours as well as in the effects of information provision (for review see Giovannoni and Rhoades [37]). However, decision-making about, and adherence to, immunotherapy with the aim of an individualised treatment in MS remains a highly complex topic.

Recently, we finished a multicentre study with 192 patients with early MS comparing group education to a stress management intervention [38]. The intervention significantly improved relevant risk knowledge and informed choice. The same applies to another recently terminated study addressing MS patients in rehabilitation clinics offering an immunotherapy group education programme [39]. In both trials, informed choices significantly increased in the intervention group (IG), but no effects on therapy decision-making or health outcomes were found.

In summary, results for EBPI and decision support indicate that it might not be sufficient to solely provide information and/or decision aids. Apparently, patients need time and support to reflect on the information and discuss options. In case of more complex decisions, for example on immunotherapy, the formerly applied approaches seem to not be sufficient, and individual decision support might be helpful in supplementing physician consultations in order to achieve successful informed SDM. In addition, group interventions are not tailored to the individual treatment decision setting and can therefore not account for differences in decision-making priorities or individual information processing.

Here, specialist MS nurses seem the ideal candidates to act as decision coaches, a concept successfully administered in other diseases [17]. Up to now only one controlled study addressed the impact of MS nurse counselling, showing beneficial effects in sexual quality of life [40].

### Aims and objectives

We hypothesise that structural changes in immunotherapy decision-making, including redistribution of tasks between specialist nurses (decision coaches) and physicians, will enhance elaborated decisions and improve healthcare management in MS. First, the intervention will empower patients to make more informed choices, tailored to their preferences and values. Second, decisional conflict will be lower compared to controls, and decisional adherence will be maintained. Third, decisional encounters will demonstrate more SDM. Finally, self-efficacy and coping competences will be enhanced.

## Methods/Design

The DEcision Coaching In persons with relapsing-remitting Multiple Sclerosis (DECIMS) trial will be carried out as a superiority cluster randomised controlled trial. Due to the nature of the intervention and the cluster design, only outcome assessment can be blinded.

A cluster design is adequate as the intervention is delivered to centres, specifically the nurses; therefore centres have to be the unit of allocation. Thus, contamination between nurses and patients of differently treated groups based on a randomisation within the centre is avoided. Moreover, it is possible to induce and observe possible structural changes in the participating MS-outpatient clinics.

Following the Medical Research Council guidance for the development and evaluation of complex interventions [41], the intervention was pre-tested with regard to feasibility and is currently piloted in two centres (St Josef-Hospital Bochum and University Medical Center Hamburg-Eppendorf). Furthermore, the main study will be accompanied by a process evaluation and an economic evaluation.

### Study setting

The study will be conducted in different neurological outpatient clinics throughout Germany. At present, 14 centres participate in the DECIMS trial (see Additional file 2 for details). The two study sites participating in the feasibility and pilot trial (St Josef-Hospital Bochum and University Medical Center Hamburg-Eppendorf) will not participate in the main study.

### Eligibility criteria

Neurological outpatient clinics in German hospitals which have a specialisation in MS are eligible to participate. Nurses are eligible if they specialise in the field of MS and are currently employed at the participating centres. Specialisation is defined as special qualifications and/or long-standing professional experience in patients with MS.

### Patient inclusion criteria

Patients older than 18 years with possible MS, defined by a typical clinical syndrome and at least one MRI lesion and/or positive oligoclonal bands [42]; and patients with relapsing-remitting MS (RRMS), according to the McDonald criteria [43], will be included. To achieve a homogeneous sample, only patients deciding on starting, stopping, or changing first-line MS immunotherapy therapy (glatiramer acetate, interferon-beta preparations, dimethyl fumarate or teriflunomide) will be included. This will lead to inclusion of recently diagnosed MS patients as well as patients under treatment, considering switching from an injectable to an oral drug. Although patients with very early or established RRMS under treatment might differ considerably with respect to attitudes, disease experience, and disability, as well as availability of therapeutic options, these factors can be controlled for and any effect of disease stage can be investigated. Likewise, these two scenarios are highly representative for daily routine and practice.

The study will use the internet for information provision and data collection; therefore only patients with access to the internet will be included.

### Patient exclusion criteria

Patients with secondary-progressive MS, primary-progressive MS, or any suspected central nervous system disease other than MS will be excluded. Furthermore, patients who are considered non-responders to a first-line treatment and who are facing a decision on escalation immunotherapy therapy (such as natalizumab, fingolimod, or alemtuzumab) or symptomatic therapy will be excluded. Also, severe cognitive deficit or major psychiatric illness affecting information uptake are exclusion criteria. In addition, patients who are related to medical personnel from the participating study centres will be excluded from the study.

### Interventions

#### Intervention group

**Figure 1:**
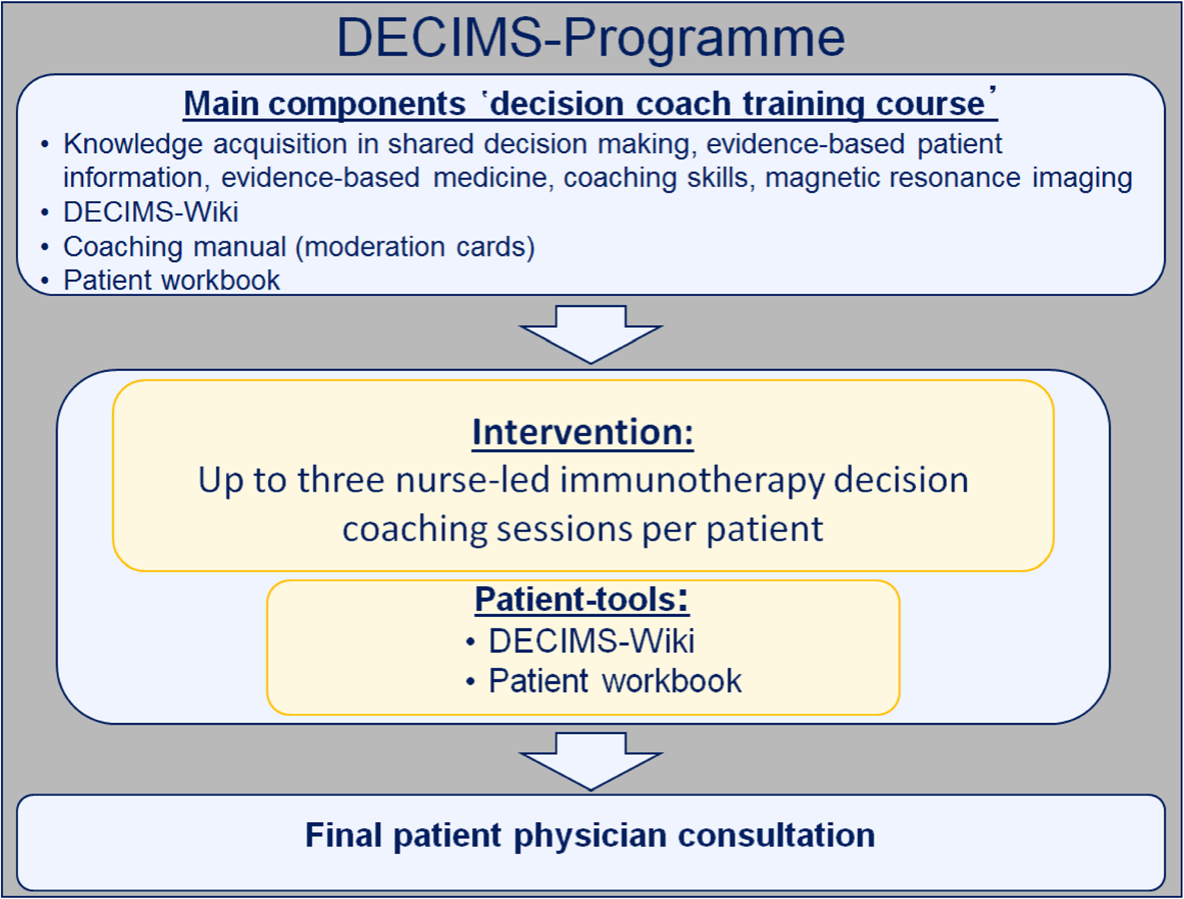
**The DEcision Coaching In persons with relapsing-remitting Multiple Sclerosis (DECIMS) trial programme.**

The ‘decision coach programme’ has been developed according to the Medical Research Council’s framework for developing and evaluating complex interventions [41]. Considering the SDM communication concept [44], nurses specialising in MS will take part in a training course to acquire relevant skills to perform immunotherapy decision coaching. Afterwards, they will conduct the study intervention, which consists of up to three decision coaching sessions per patient. As part of the intervention, a web-based information tool, the DECIMS-Wiki, moderation cards, and a patient workbook have been developed to provide information and to give guidance throughout the decision-making process (see Figure 1).

The DECIMS-Wiki has been developed based on literature searches and an update of available brochure-based information materials from previous studies [45]. The tool was drafted by the research group at the study centre in Hamburg and will be continuously revised in cooperation with all participating study centres. In addition, each patient will be provided with a patient workbook, which is targeted to the specific kind of decision to be made (first treatment or switchers). The decision nurses are instructed to organise the coaching process considering six subsequent topics to be discussed in a decision-making process [46]. The six steps of an SDM-process are:

1. to review the problem requiring a decision-making process;
2. key message: decisions cannot be made based on evidence alone. It is the patient who needs to decide;
3. information about pros and cons of each option (including no immunotherapy);
4. expectations of the patient;
5. decision (progress in decision-making, deferment is a possible decision); and
6. arrangements.

The moderation cards and the patient workbook are structured according to the above described six SDM steps. Further, the moderation cards guide the inclusion and connection of the DECIMS-Wiki and the patient workbook into the coaching process.

The curriculum of the training programme is based on previous expertise in the training of consumer representatives [47]. Moreover, train-the-trainer expertise from a previous programme was used [48]. The training focuses basic skills in SDM, including EBPI and coaching, using methods established in physician communication trainings [49,50]. The training includes further guidance on using the DECIMS-Wiki and insight into the use, interpretation, and impact of findings on magnetic resonance imaging (MRI) scans.

After randomisation, nurses in the intervention clusters will receive special training. All nurses will receive the same training provided by the same research team. The training consists of provision of preparatory materials and tasks, a training course (three days, 16 hours in total), and a structured feedback (via telephone) concerning coaching performance in practice after the training course. Knowledge gain of the nurses will be evaluated through questionnaires (before and after training). Up to six coaching sessions per decision coach will be video-recorded shortly after the training to give structured feedback on coaching performance. The videos will be evaluated independently by two researchers who will use standardised forms to assess the quality of the coaching session (in terms of SDM, EBPI, and coaching competencies). When nurses, do not implement important aspects which ensure a standardised delivery of the intervention despite receiving this feedback, they will be excluded from the study. Those aspects are:

1. no coaching according to the SDM criteria,
2. no use of the DECIMS-Wiki during the coaching,
3. not able to explain the bar charts on treatment effects to participants,
4. no appropriate use of the moderation cards or their contents during the coaching.

However, before a nurse will be excluded from the study, efforts will be taken to communicate that information (for example through extra training).

Eligible patients will receive their first coaching session with the decision coach within two weeks after inclusion with up to three coaching sessions per patient. Periods between sessions should not exceed two weeks. A single coaching session will last up to one and a half hour. Patients and decision coaches (nurses) will evaluate the coaching sessions via web-based questionnaires. Additionally, decision coaches will keep a logbook to document each coaching session.

Patients will be given access to the DECIMS-Wiki to prepare for coaching sessions, to gain further relevant knowledge, and to be able to reflect upon options between coaching sessions. After the final coaching session, patients will see a physician within two weeks to decide upon immunotherapy. It is possible that in individual patients more than one medical encounter will be necessary in order to make a decision. A total of 40 physician-patient encounters will be audiotaped in four centres in order to measure possible changes in the physician-patient communication (for detailed information see process evaluation).

In addition, physicians in both groups will receive an information package on SDM. The package consists of the following information:

1. A letter, including information about the study, the SDM concept, and the request to follow the SDM concept during the study.
2. A link to a video (password protected), which shows a physician-patient conversation according to the SDM concept.
3. An article, which provides information about SDM in the field of neurology [51].

This information will be handed out to all participating physicians in the IG as well as in the control group (CG), since it is intended to assess the effects of the decision coaching intervention using trained nurses alone.

### Control group

The CG will be given access to the evidence-based online patient information-tool (DECIMS-Wiki), which will also be used in the IG, including an information sheet on how to use it, and otherwise receive care as usual.

Offering both groups access to evidence-based information will allow for a better estimate whether possible differences between groups can be attributed to nurse-led decision coaching. For the same reason, physicians in the CG also receive the SDM package.

### Criteria for discontinuation

#### Adverse events

Our previous work has shown that even complex information about MS treatment evidence is appreciated by patients [32]. Handing over information provision from physicians to nurses might induce concerns among MS patients. However, the framing of the intervention is as ‘preparation for a medical encounter’, therefore, we do not believe that patients perceive the intervention as a reduction of physician attention. The process is individualised to the decision pace of individual patients, allowing for individual decision-making processes. To account for possible adverse events, we will continuously monitor satisfaction with the process, which will be also communicated to the Data and Safety Monitoring Board (DSMB). We do not foresee any other harm of the intervention.

#### Patient withdrawal

Patients in both groups can quit the study at any time point. Patients who withdraw from the study are asked whether they agree to continue to fill in a limited set of questionnaires related to the primary study outcome.

#### Physician encounters

It is aimed that patients do not see a neurologist during the coaching stage. However, there are situations where patients have to or want to see a neurologist (for example, for relapse management). In these cases, neurologists in the participating outpatient clinics and practises are asked whenever possible not to discuss immunotherapy options. Still, this might not always be appropriate and some patients might also consult a practice-based neurologist. Any physician encounter will be documented.

### Strategies to improve adherence

#### Decision coaches

All decision coaches will receive a study coach folder including all relevant documents of the training, the patient workbooks, moderation cards, and further material on communication and coaching.

Coaching fidelity will be secured through different measures: first, an interactive three-day training course in Hamburg; and second, video feedback of two coaching patients per nurse in the respective centre. Also, they will be contacted regularly (monthly during the first three months and every two to three months afterwards) to ensure quality standards of coaching sessions and support the decision coaches. Calls will consist of open and closed questions and decision coaches will have the opportunity to come up with their own aspects (as for example questions concerning coaching procedures or the DECIMS-Wiki). Furthermore, we aim to hold three to four telephone conferences per year with participating nurses from the IG. This will provide an opportunity for the nurses to connect and share experiences, for example to discuss difficult coaching situations.

#### Logbook

Decision coaches are further asked to use an online logbook for each participant to support a standardised delivery of the intervention.

#### Coaching sessions

Moderation cards will be provided to decision coaches to ensure that the key components of the intervention are delivered to the patients. This adds to the patient workbook, which also provides guidance through the SDM steps. Coaches might prepare sessions by looking into the coaching cards. In each coaching session it is aimed that the DECIMS-Wiki, the moderation cards, and the patient workbook are used. Moreover, the workbook and the moderation cards do serve as structuring aids for the encounters.

#### Strategies to facilitate the utilisation of the DECIMS-Wiki

Decision coaches will be informed about the DECIMS-Wiki and use the tool during the training course, and the DECIMS-Wiki will be addressed during telephone calls and in the logbook. Beyond that, decision coaches will be informed when the platform has been updated.

### Patients

If patients miss an appointment, they will be contacted by the decision coach to arrange a new appointment. Patients will be contacted by email by a member of the coordinating centre in Hamburg when it is time to fill in a form, and will be asked to complete the questionnaires within a specified time period. Patients who miss the completion will again be reminded by email and telephone. When appropriate, patients will be asked to fill in a questionnaire in the outpatient clinic directly after an encounter.

Decision coaches will inform patients about the DECIMS-Wiki and use the tool during the first coaching sessions reminding patients to use it between sessions. All patients will receive a personal password for the DECIMS-Wiki and an information leaflet about the tool.

### Relevant concomitant care

#### Relapse management

In case of deterioration, for example a relapse during the coaching stage, the participant is free to consult a specialist and receive appropriate treatment.

### Outcomes

For a list of the major endpoints of the DECIMS trial, see Table 1.

**Table 1:** **Major endpoints CRCT**

**Instrument**		**Measurement time points**						
		**Enrolment**	**Allocation**	**Post-allocation**				
		**-t**_**1**_	**t**_**0**_	**t**_**1**_	**t**_**2**_	**t**_**3**_	**t**_**4**_	**t**_**5**_
Eligibility screen		X						
Informed consent		X						
Allocation								
Sociodemographic data		X	X					
EDSS			X					
SDMT			X					
MS-related data and resource use			X				X	X
MMIC:								
	Risk knowledge		X			X	X	X
	Attitude			X	X	X		
	Immunotherapy status		X			X	X	X
Dyadic DCS				X (nurse)	X (physician and patient)			
Dyadic MAPPIN’SDM				X (nurse and patient)	X (physician and patient)			
HCR trust scale (Physician/Nurse trust)				X	X			
PBMS			X			X		X
CPS			X			X		X
Decision autonomy						X	X	X
CSES			X			X		X
HAQUAMS			X			X		X
HADS			X			X		X

t_1_ = after last decision coaching; t_2_ = directly after final physician decision encounter; t_3_ = two weeks after final physician encounter; t_4_ = three months after final physician encounter; t_5_ = six months after final physician encounter. CPS: Control Preference Scale; CSES: Coping self-efficacy scale; DCS: Decisional Conflict Scale; EDSS: Expanded Disability Status Scale; HADS: Hospital Anxiety and Depression Scale; HAQUAMS: Hamburg Quality of Life in MS Scale; HCR trust scale: Health care relationship trust scale; MAPPIN’SDM: Multifocal Approach to Sharing in Shared Decision Making; MMIC: Multi-dimensional measure of informed choice; MS: Multiple Sclerosis; PBMS: Planned Behaviour in MS Scale; SDMT: Symbol Digital Modalities Test.

### Primary outcome

We have previously applied the multi-dimensional measure of informed choice in two controlled trials [52]. Here, informed choice is defined as a compound measure combining three dichotomous measures: risk knowledge, attitude, and therapy uptake. Informed choice encompasses adequate risk knowledge, with either uptake or non-uptake of immunotherapy, and a corresponding (congruent) positive or negative attitude. Attitude will be assessed using a single question directly after the final physician encounter. Uptake will be evaluated from the patient after six months. Risk knowledge will be measured using a previously developed and adapted questionnaire 14 days, and three and six months after the last physician encounter [53]. As applied in a previous trial, the cut off for adequate risk knowledge will be defined *a priori* as the value that 30% of all patients with highest scores reach at baseline. In addition, risk knowledge will be analysed as a continuous variable to enable comparability with other studies. Earlier trials have shown that patients who meet the primary endpoint more often realise their preferences [38,39].

### Secondary outcomes

The Decisional Conflict Scale ((DCS) [54]) has been used in numerous decision support interventions and is regarded as a tool to monitor comfort with the decision process. Here, a dyadic DCS [55] (patient - decision coach and patient - physician) will be applied as key secondary endpoint after the last coaching session (IG) and after the final physician encounter (for both the IG and CG).

Further tools will be used to monitor decisional processes assessing autonomy preferences (Control Preference Scale (CPS) [56]), behavioural beliefs, and self-efficacy (Planned Behaviour in MS Scale (PBMS) [57]). Coping and self-efficacy will be assessed by application of the recently validated Coping Self-efficacy Scale (unpublished data Pöttgen J, Mohr DM, Ziegler K, Gold SM, Heesen C) based on Chesney *et al*. [58]). Perceived involvement in coaching and decisional encounters from patients’ as well as physicians’ and nurses’ perspectives will be evaluated with the Multifocal Approach to Sharing in Shared Decision Making (MAPPIN’SDM) evaluation [59]; applying a newly developed short version. We will assess participants’ trust in nurses and physicians [60].

Decisional adherence (including the decision against immunotherapy) and acceptance of the intervention will be assessed from patients using a standardised questionnaire at three and six months after the last physician encounter (for both the IG and CG). Finally, duration of decision coaching and physician encounters will be documented.

### Tertiary outcomes (control and safety parameters)

As control parameters we will use measures for anxiety and depression using the Hospital Anxiety and Depression Scale ((HADS) [61]), and disease-specific quality of life using the Hamburg Quality of Life in MS Scale ((HAQUAMS) [62]). Moreover, standard disease-monitoring parameters will be obtained; relapses and disability as measured by Expanded Disability Status Scale ((EDSS) [63]) and the Symbol Digital Modalities Test ((SDMT) [64]) for cognition. Occurrence of relapses will be evaluated at baseline, 14 days, and three and six months after the last physician encounter (for both the IG and CG) using a standardised questionnaire.

### Health economic outcomes

Data to perform health economic analyses will be assessed with an adapted tool used in a previous trial [35]. Patients will be asked to consent for collection of health insurance data for the study period.

Focus will be the rate of patients initiating MS immunotherapy as well as relapse treatment prescription (including route of administration). Further, number of MS-related visits to neurologists and general physicians, number of MRI scans, missed days at work, and hospital stays will be evaluated.

### Participant timeline

**Figure 2:**
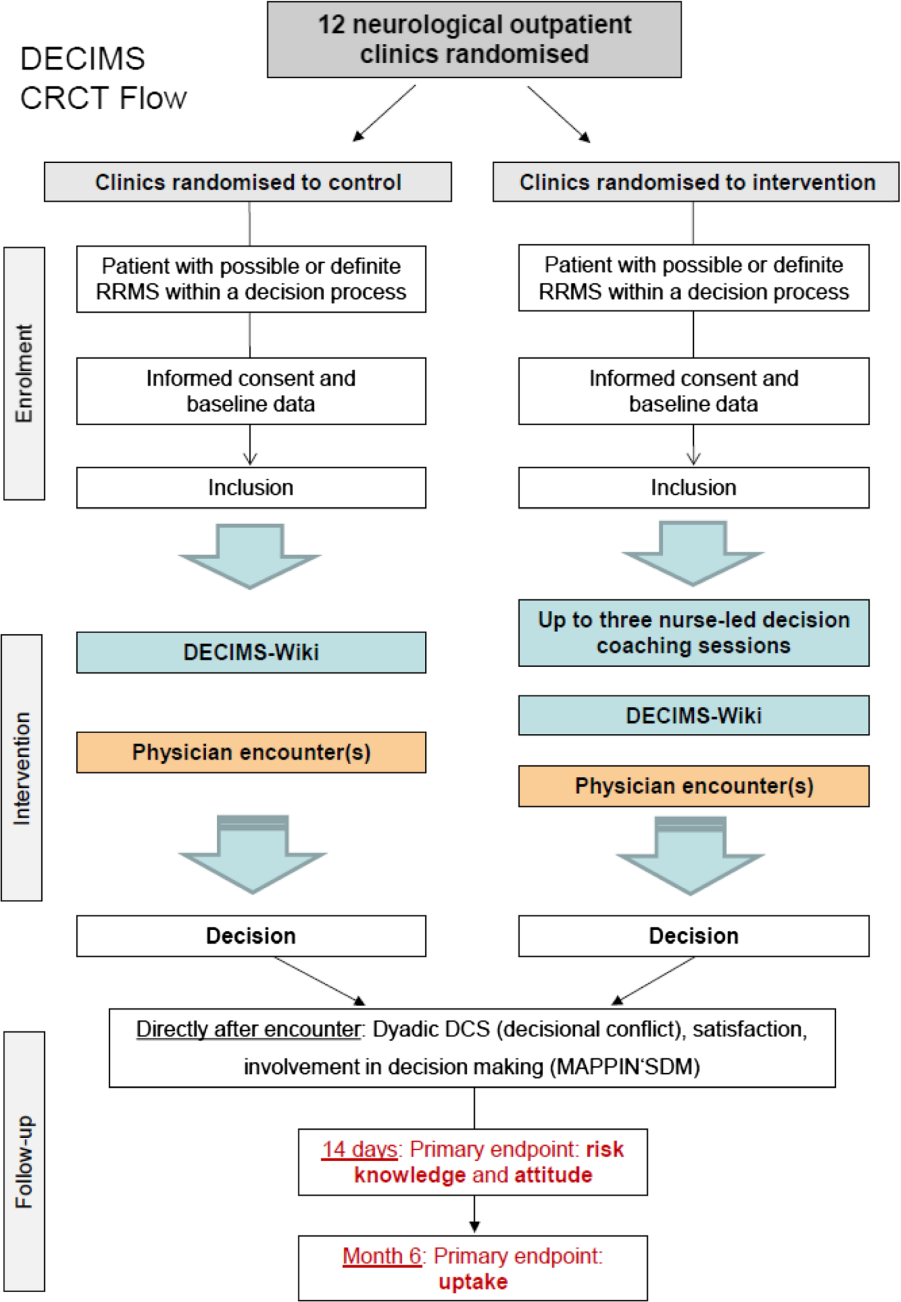
**DEcision Coaching In persons with relapsing-remitting Multiple Sclerosis (DECIMS) trial flow.**

CRCT: cluster randomised controlled trial; DCS: Decisional Conflict Scale; MAPPIN'SDM: Multifocal Approach to Sharing in Shared Decision Making; MMIC: Multi-dimensional measure of informed choice RRMS: relapsing-remitting multiple sclerosis

For a description of the flow of the DECIMS trial see Figure 2.

### Screening visit

As it is not possible to coach all suitable patients in every participating centre, not all potentially eligible patients will be included. To avoid selection bias, possible recruitment days will be randomly determined by a statistician for those centres, and an independent person will call the study sites weekly to inform them about the recruitment day(s).

Nurses (for both the IG and CG) will create a list on all recruitment days, recording all MS patients who attend the outpatient clinic that day. Potentially eligible patients will be identified using a screening form (form one) during an appointment. Screening form one has to be filled in for every patient. Therefore, reasons why patients are not suitable will be documented as well. When patients seem to be suitable for the study, they will receive information about the study from the physician or from a nurse. For diagnostic cases in which an early treatment will be discussed, physicians will invite patients after having communicated diagnostic test findings. In the case of treatment switchers, the encounter is stopped before counselling about the possible immunotherapy options will take place. Physicians have to fill in a second screening form, including the study inclusion criteria, for all patients who seem to be suitable for the study. Informed consent will be obtained from patients fulfilling the inclusion criteria after they have had enough time to read the study information sheet and ask questions. The encounter will be stopped when informed consent is given, and patients will be invited to fill-in baseline data via an online questionnaire database and will receive an access code to the DECIMS-Wiki after the completion of baseline questionnaires. Depending on the cluster’s group allocation, patients will receive a new appointment with the physician or an appointment with the decision coach. Suitable patients, who are not willing to participate in the study, will be asked for the reason (screening form two).

### Baseline data and allocation

After information about group allocation, patients in the CG will receive an information sheet about the DECIMS-Wiki from a nurse, will receive usual care, and a decisional encounter with the physician will be scheduled. In the IG, apart from information about access to the DECIMS-Wiki, an appointment for a first encounter with the decision coach will be scheduled within 14 days. After inclusion and the completion of baseline questionnaires, patients will receive an electronic access code to the DECIMS-Wiki, which is linked to the information technology platform of the *Krankheitsbezogenes Kompetenznetz Multiple Sklerose* ((KKNMS) Competence Network Multiple Sclerosis). Further appointments will be planned at the end of each encounter, which could be up to two more with the decision coach and up to two with a physician.

### Encounters and web-based visits

After the last encounter with the decision coach, prompt feedback from patients will be collected at the centre by web-based questionnaires. After up to three meetings with the decision coach (visits one to three), up to two decisional encounters with a physician will take place within four weeks. Decision coach encounters will be videotaped and sent to the Hamburg study centre for analysis by the research group.

Patients will be followed up on using web-based questionnaires within 14 days, after the final encounter with the physician (web-based visit), after three months (web-based visit), and after six months (web-based visit and standardised telephone interview).

### Additional visits

At least three randomly selected patients from each intervention cluster will be contacted after the follow-up period and asked to take part in an additional interview, which will be conducted in the context of the accompanying process evaluation (for details see process evaluation). Furthermore, when additional funding is provided, patients will be contacted via telephone by the study centre to assess their current treatment status after 12 and 24 months.

### Sample size

The primary endpoint of the DECIMS trial is informed choice, that is, a fitting of good knowledge, a given attitude, and the corresponding uptake. Each of these three dimensions will get a dichotomous rating of ‘yes’ or ‘no’. Based on data from prior studies [35,36], we assume that after the intervention, 60% of patients in the IG will show ‘adequate’ knowledge compared to 40% in the CG. Adequate knowledge is defined as the number of questions correctly answered by 30% of patients at baseline, which was also applied in previous work. We assume that in the IG group about 80% of attitudes and decisions are congruent, compared to 70% in the CG. Therefore, we expect 48% of IG patients to make informed decisions compared to 28% of patients in the CG. In order to detect this difference with a power of 90% and a significance level of alpha = 0.05, 12 clusters with 23 patients per cluster will be needed, assuming an intra-cluster correlation coefficient of 0.0045, which is a conservative estimate based on data from our previous trial [38].

Assuming a dropout rate of 10%, 25 participants per centre will be needed, accounting for a total of 300 participants in 12 clusters. In all our previous trials on EBPI, loss to follow-up was less than 10%. Therefore, 10% seems a realistic and conservative assumption.

### Recruitment

Contact persons of different MS clinics in German hospitals were contacted by the project leader (CH) and informed about the study. All outpatient clinics which were willing to participate have been included in the study. Recruitment strategies will be individualised to ensure that centres’ specific requirements are addressed (please see screening visit). The feasibility of recruitment is currently being tested in the pilot study.

### Allocation

Clusters will be stratified by type of hospital (university hospital or community based hospitals). Allocations will be computer generated and will be performed by a statistician not involved in the conduct of the trial. Prior to randomisation of the centres, contextual factors of the participating centres will be assessed in a baseline survey.

Centres will be aware of their allocation status. To minimise selection bias, patients will not receive explicit information about their allocation group, but will only be informed that they will be assigned to one of two methods of information provision about MS immunotherapy (information provision only or information provision plus information by a nurse).

### Blinding

Blinding of patients in patient information trials is difficult as the intervention can be easily detected. Therefore, due to the nature of the intervention it is not possible that clusters and patients are blinded. Nevertheless, contamination is avoided by the cluster design and patients will only be informed that two different ways of decision support regarding immunotherapy, information provision only or information provision plus information by a nurse, will be assessed. Assessment of the endpoints will be evaluator blinded as persons concerned with outcome assessment (by telephone interviews) will not be informed about patient and centre allocation.

### Data collection methods

Data will be collected at seven time points using web-based questionnaires (see Table 1). Use of the web platform will be explained via information sheets and through personal information within the study centres. Additionally, some data will be collected by telephone using trained and blinded interviewers after six months and, depending on funding, after 12 and 24 months (see Additional file 1).

### Statistical methods

For the primary outcome measure, the proportion of informed decisions within a treatment group, a generalised linear mixed model, reflecting the hierarchical structure of the data will be used [65]. Due to the relatively small numbers of clusters, imbalances in baseline characteristics on cluster and individual level may occur which are not fully covered by randomisation. Therefore the model will be adjusted for baseline variables. The treatment effect will be analysed at cluster level, whereas covariates will be analysed individually by the model. For the secondary outcome measures linear mixed models or generalised linear mixed methods will be used adjusting for clusters by random effects. These models also allow analyses of subgroups. All analyses will be performed on the intention-to-treat population.

It is planned to perform subgroup analysis of the two groups of patients included in the trial: first, those with a recent diagnosis, facing an initial decision on immunotherapy and second, those considering changing to an oral treatment. Apart from demographic baseline data, all analyses will be cluster-adjusted. We will report causes for study withdrawal for each patient to clarify whether there are any differences between the intervention and control clusters.

In addition, a sensitivity analysis will be performed to evaluate the robustness of study results and to explore different imputation techniques. Altman [66] addressed that there is no ideal method to address missing data. Therefore, different common imputation techniques [67] will be applied and reported with as well as without imputation techniques as suggested by Altman [66]. Last observation carried forward, as well as best and worst case scenario for dichotomous outcomes and multiple imputation techniques, will be conducted in the sensitivity analysis [68].

### Harms

As relevant adverse events are unlikely, no interim analyses are planned and no stopping rules will be applied. Nevertheless, safety measures are applied as tertiary endpoints to control for anxiety, depression, and disease-specific quality of life. Furthermore, standard disease monitoring parameters will be collected (such as relapse rate, disability status, and functional status).

### Research ethics approval

Ethical approval has been obtained from the ethical committee of Hamburg Chamber of Physicians (approval number: PV4576), and has been obtained from local committees at each centre location. Please see Additional file 2 for details.

### Feasibility study and pilot trial

The intervention and the study procedures including outcome assessment were pre-tested through a feasibility study and are currently being tested in a subsequent pilot randomised controlled trial in the study centres in Hamburg and Bochum. The pilot study aims at first testing the randomisation procedure and second to gather data on feasibility of conducting the main trial.

For the feasibility study, four nurses specializing in MS from the centres in Hamburg and Bochum have received training in Hamburg. The feasibility study has been conducted over six months and 12 patients were included. Each decision coach has coached three patients, chosen by either the decision coach or the physician. The feasibility study aimed to evaluate the training course, access the acceptability of the programme (decision coaches, patients, and study sites), and to detect barriers and facilitators. Therefore, telephone interviews with included patients were conducted and analysed.

Currently, a pilot randomised controlled trial is being performed in the two centres in Hamburg and Bochum. Here, we aim to recruit 30 patients per centre, following the main study procedure with the following adaptation: both intervention and control intervention will be tested in each study centre. Therefore, both centres will receive randomised days to recruit either for the IG or CG.

Both the feasibility and pilot study follow the main hypothesis that the concept is feasible for decision coaches and patients. In detail, it is tested whether:

1. patients agree on initially consulting a nurse (decision coach),
2. the patient workbook is acceptable for patients and decision coaches,
3. the DECIMS-Wiki is helpful in the decision process,
4. the patient workbook and information platform can be used together during encounters,
5. study recruitment is feasible, and
6. outcome measurements are acceptable.

Data from the feasibility study have been used to adapt the train-the-trainer course to nurses needs in the encounters and we developed moderation cards (instead of an information sheet) for the decision coaches. Further, as a result of the pilot study, it has been decided to videotape all coaching sessions.

In addition, different possibilities to present data of risk communication (for example graph or pictogram) will be evaluated in terms of knowledge and understanding via web-based surveys in cooperation with the German MS Self-help Society (DMSG). For example, an education tool to support the comprehension of confidence intervals will be tested.

### Process evaluation

Process evaluations should generally be accompanying complex intervention studies in order to measure programme fidelity and explore reasons for an effective or ineffective intervention [69]. Following the guidance of the Medical Research Council, the cluster randomised controlled trial will be accomplished by a process evaluation in order to assess study processes concerning patients, decision coaches, and the setting and context of the study. A process evaluation is of great use to understand the results of a study, and to later translate a successful intervention into practice [41,70].

Recently, Grant *et al*. [70] have published guidance for the development of process evaluations specifically addressing process evaluations for cluster randomised controlled trials of complex interventions. This framework will be used to guide the process evaluation of this study.

Ferlie and Shortell [71] suggest four levels of change which have to be considered in order to reach quality improvements in health care systems: individual level, group or team level, overall organisation level, and larger system level or environment in which individual organisations are embedded. Thus, teams build an important basis for changes. Depending on the level(s) and the intervention targets, different theories are relevant [71]. The intervention in this project targets people with MS, who face a decision) concerning immunotherapy (begin, start or change of immunotherapy). Therefore, MS nurses who work in an outpatient clinic will be trained as decision coaches. The study intervention affects all persons who are involved in the decision-making process; patients, physicians, and nurses. Presumably, a successful intervention depends on the support and attitude of the whole MS outpatient team towards the planned decision coaching intervention. However, it is hypothesised that a successful implementation of the intervention relies decisively on the motivation and attitude of the trained MS nurses.

The knowledge, which will be imparted during the nurse training course, is based on the principles of evidence-based medicine [72], and the knowledge transfer reflects established educational theories and concepts [73,74]. Further, the theory of planned behaviour [75] has been applied concerning contents of the training and the transfer of knowledge into practise (decision coaching performance).

Overall, the project is guided and determined by the principles of evidence-based medicine [72] and EBPI [76]. Further guidelines and concepts are considered: the MRC guidance for developing and evaluating complex interventions [41] for the design of the study and the SDM concept [46] to design and conduct the decision coaching intervention.

As mentioned above, the process evaluation is a mixed-methods study [77]. Qualitative and quantitative methods will be applied in combination and will be analysed together in order to illustrate and explore changes related to the decision coaching intervention on the cluster level (as for example change of structure in the outpatient’s clinics), as well as the individual level (as for example attitudes of the nurses). Partly, the quantitative results of the trial will be used to determine questions of the qualitative interviews to be conducted after the study. Therefore, quantitative and qualitative methods are used intentionally to acquire a comprehensive impression of study processes and mechanisms.

In this process evaluation, a variation of the embedded design of mixed-methods studies is applied [78]. Besides, qualitative methods have been used within the feasibility study before the start of the trial to investigate study materials (the DECIMS-Wiki and patient workbook) with regard to user-friendliness and comprehensibility (see also trial protocol).

**Figure 3:**
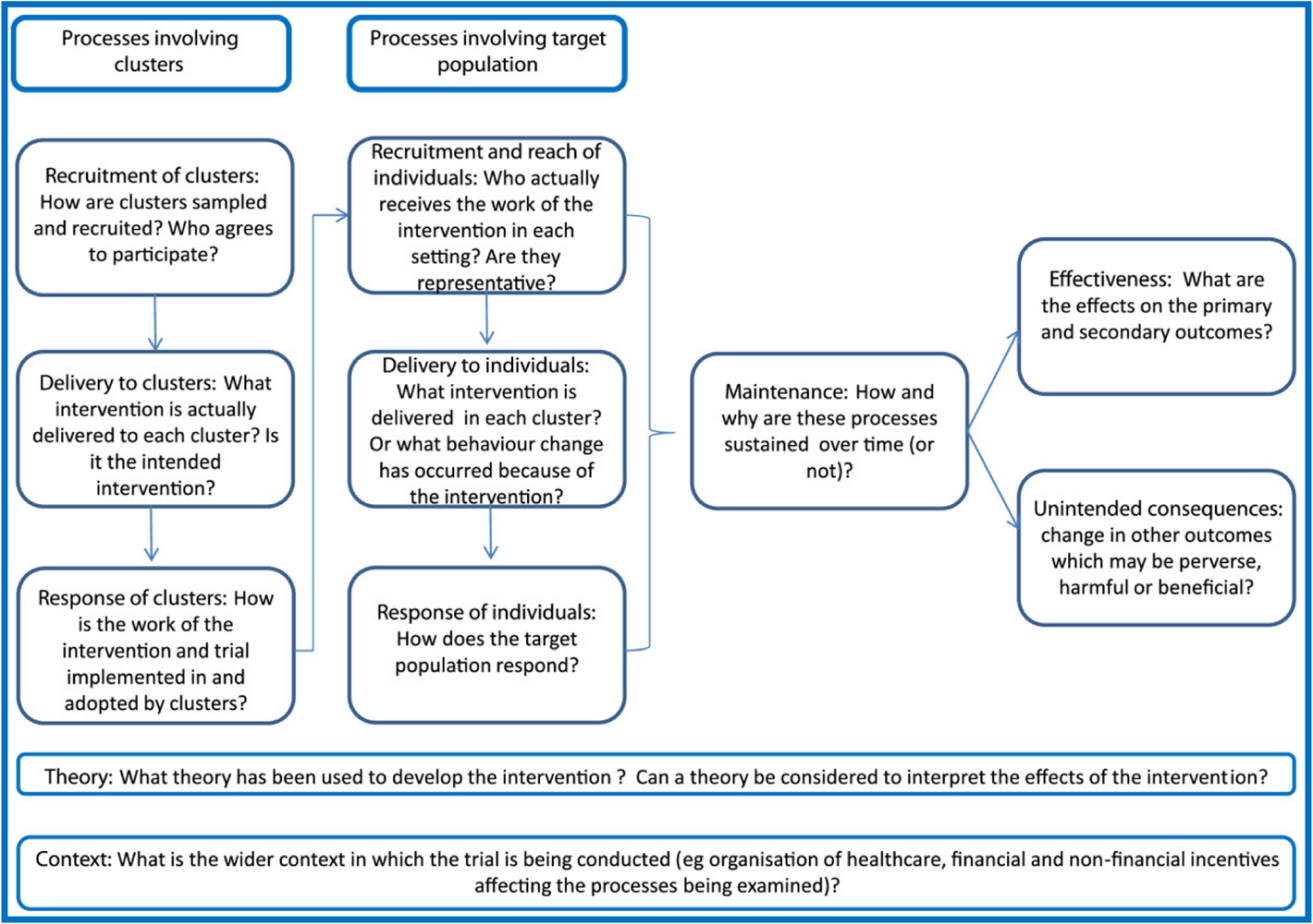
**Overview of the process evaluation steps (from Grant** ***et al*** **. [** 70 **]).**

The framework proposed by Grant *et al*. [70] consists of 10 domains (Figure 3). Three domains are comprised of processes in which clusters are involved: recruitment of clusters, delivery to clusters, and response of clusters. Three domains address the processes within the target population: recruitment and reach in individuals, delivery to individuals, and response of individuals. Further chapters cover theory, context, maintenance, and unintended consequences (see Figure 3).

As shown in Figure 3, effectiveness is displayed additionally to the 10 process evaluation steps. This concerns the results of the trial, which for instance determine the research questions of the qualitative interviews with patients after the trial is finished.

The primary aim of this theory based process evaluation is to explore underlying mechanisms and to determine effect modifying factors. Following the framework, the objectives are to:

1. explore the reaction of the clusters (such as the delivery of the intervention, response to the training course, and maintenance);
2. identify barriers and facilitators concerning the delivery of the intervention (coaching) to the patients;
3. assess cluster-specific differences (such as cluster reach and organisational differences);
4. measure the reaction of individuals with respect to responsiveness towards study recruitment and the intervention;
5. identify barriers and facilitators of study participation and of study retention;
6. ascertain structural problems;
7. analyse which study components work or do not, and for which reason; and
8. look for unintended consequences of the intervention (decision coaches, patients, and clusters).

Additional file 3 shows the application of the framework to this study. In the following, the planned components of the single domains are described in more detail. Questionnaires, which will be used for this process evaluation, have been developed by the research team and were tailored to the intervention. The questionnaires have been tested for usability. Nonetheless, published work in this field has provided useful guidance for the development of the questionnaires for nurses [79]. Most of the described content of this process evaluation refers to the IG. Content which also refers to the CG is marked (CG).

### Context

Relevant factors of the German health system will be described and their relevance for this project will be discussed. A total of 14 different MS outpatient clinics are involved in the study, nine related to university hospitals and five to community based hospitals. Depending on the location, there is significant variation between the outpatient clinics, and due to factors such as size, practice hours, and clinical focus of the clinics, the number of potential study participants vary considerably. In the planning phase of the study, participating centres were visited in order to gain important information about structure and processes within the outpatient clinics. Based on the evaluation of this information, the conduct of the study and process evaluation content was adapted.

Patient populations of the different outpatients clinics consist of patients who:

1. recently received a diagnosis and require information about therapy options,
2. visit the outpatient clinic regularly and now face a treatment decision (start, stop, or change of immunotherapy),
3. seek a second opinion, and/or
4. other (for example acute relapse).

Accordingly, the staffing (doctors, study nurses, MS nurses, and receptionists) and the roles of outpatient clinics in the respective neurological department are organised differently. Similarly, the clinics differ in terms of everyday processes, the functions assumed (by doctors and nurses) in patient care, and patient populations. Many of these differences have been noticed during the visits. As a result, the analyses of these different contextual conditions will provide important information concerning transferability of the concept to MS outpatient clinics in Germany, and to other countries and medical fields.

Prior to randomisation of the centres, study contextual factors will be assessed in a baseline survey (self-developed questionnaire (for both the IG and CG)). In addition, certain aspects of possible centre-specific effects (promoting factors and barriers) will be explored in more detail after completion of the study through qualitative interviews. For example, the possible advantages and disadvantages of the team structure (such as number and qualification of employees) for the work of decision coaches will be studied by qualitative interviews.

### Recruitment of clusters

Clusters consist of participating MS outpatient clinics (see above). Within the collection of baseline data, it is planned to ask the physicians and nurses within the centres why they are participating in the study. Centres that withdraw participation will be asked for the reason.

### Delivery to clusters

The decision coach training, which one or two MS nurses from all intervention clusters will receive, has been developed and will be performed by the study working group in Hamburg. In order to better understand and interpret modes of action of the complex intervention, a feasibility study, followed by an ongoing pilot study, was performed. Based on the results of these studies, both the training of nurses and the intervention (coaching sessions and supplementary materials) have been revised.

The intervention leads to changes in common practice within intervention clusters. On the one hand, MS nurses get involved in a new or expanded field of activity and acquire the relevant skills for this through the decision coach training. In addition, the local structure in MS outpatient clinics is changed due to the implementation of the coaching concept.

As part of the process evaluation it is determined to which extent clusters have received the intervention. The focus of the observation is to evaluate whether the intervention has been conveyed to all clusters in the same way. For this purpose, it is documented whether all participating nurses attend all training lectures, including subsequent training activities. Important aspects related to the training are assessed at the end of the training using questionnaires covering, for example, satisfaction and understanding. A knowledge assessment on relevant training content is performed before and after the training.

Further, it will be measured whether the course of the study (for example recruitment) has been communicated to all outpatient clinics (intervention and control clusters). Moreover, it will be captured whether physicians have received the SDM information, and if at least all principal investigators participated in the web-based meeting where the initiation of the DECIMS trial at the centre was performed (for both the IG and CG).

### Response of cluster

An important part of the process evaluation is the attitudes of stakeholders (doctors and nurses) about the intervention and related structural changes. Quantitative surveys will be conducted at two time points (outpatient clinic teams in the intervention and control clusters) and at five time points (decision coaches: baseline, after training, after six weeks, six months, and after study completion) to determine changes in the course of the study (see Additional file 4).

In addition, physicians and nurses in the intervention clusters are interviewed after study completion to determine whether attitudes have changed during the course of the study and, if so, what factors have led to these change. Interviews will be semi-structured [80] and are subsequently evaluated by content analysis [81].

Further, it will be evaluated whether there are any changes in the professional relationship between nurses and physicians due to the intervention. Besides possible changes in the professional relationship between physicians and nurses, changes in the physician-patient communication will be addressed. Therefore, in four centres 10 physician-patient encounters will be audiotaped and analysed concerning SDM content (MAPPIN’SDM).

An important aspect is the implementation of the intervention in different centres and to determine characteristics of centres that determine successful implementation (barriers and facilitators). For example, the number of patients within centres or the qualification of the MS-nurse might be important factors here.

Apart from interviews with decision coaches, facilitators and barriers of standardised implementation of the decision coaching will be assessed through a nurse logbook for each patient. In this web-based logbook, nurses record important information about patients and coaching appointments, such as duration or discussed SDM steps.

Following the training, the decision coaches perform training coaching sessions with two patients, recorded on video. As mentioned above, these first coaching sessions are evaluated and nurses receive telephone feedback after every patient by AR, together with a psychologist.

Willingness of nurses to work and further train in the new action field, use of the distributed materials (moderation cards and patient workbook), use of the DECIMS-Wiki, and gathering information beyond the provided information are also an important part of the process evaluation and will be assessed through logbooks, questionnaires, and qualitative interviews after study completion.

Some evaluation questions are based on the theory of planned behaviour [75] and aim to determine factors for a good immunotherapy coaching. Good immunotherapy coaching, as defined in the study, is provided when all six SDM steps have been addressed. Therefore, all coaching sessions will be videotaped and we aim to analyse the videos of at least 50 randomly chosen patients (dyadic MAPPIN’SDM evaluation [59]). Upon completion of the study, questions which arise from the video analysis and quantitative evaluation are addressed through qualitative interviews. In addition, it will be assessed by questionnaires whether and to what extent the intervention has had an impact on nurses in the intervention clusters who did not receive the training. In the following, a selection of aspects that will be covered is listed for the physicians of the IG:

1. attitude towards the intervention,
2. distress through additional organisational effort,
3. reduction of workload due to nurses’ counselling,
4. handing over responsibility to nurses,
5. change in patient communication, and
6. change in communication with nurses.

A selection of aspects that will be covered is listed for the decision coaches of the IG:

1. attitude towards the intervention and personal interest,
2. higher workload versus work routine,
3. changes in the inter-professional relationship to the physicians and others, and
4. facilitating factors and barriers.

### Recruitment and reach in individuals

To ensure a standardised recruitment, the recruitment procedure was determined after most of the participating centres had been visited by members of the research team. A non-responder analysis will be conducted in all centres. On the one hand it should be ascertained whether there are fundamental differences between control and intervention centres. On the other hand it should be determined if there are considerable variations in the reasons for or against study participation in individuals. Therefore, patients will be briefly asked for their reason/s not to take part in the study (screening form two). Moreover, reasons for taking part in the trial will be surveyed.

### Delivery to individuals (dose delivered)

As aforementioned, all coaching sessions will be videotaped and analysed. The analysis focuses the assessment of coaching quality on respective SDM content Here, patient information about benefits and harms of therapy options, using the DECIMS-Wiki, are of particular relevance. In addition, nurses document in the logbooks which SDM steps have been discussed during the coaching session, how many coaching sessions have been performed, and duration of sessions. Patients are asked to fill in a short questionnaire directly after the last physician encounter. The questionnaire assesses, among other things, the use of and satisfaction with the DECIMS-Wiki, especially focussing on nurses as a possible influencing factor. For instance, the attitude of the nurse towards the intervention could have an impact on coaching performance.

After study completion, three patients per IG centre (purposeful sampling) will be questioned, using semi-standardised interviews, in order to determine which aspects of the intervention were helpful for the patient in the decision-making process, and where any action or change was needed. The interview guide will be created based on the analysis of the questionnaires. Depending on resources, use of the DECIMS-Wiki will be evaluated in the CG.

### Response of individuals (dose received)

Apart from monitoring the transmission of the intervention, patients’ responses will be investigated, focussing on:

1. changes in risk knowledge (using the risk knowledge questionnaire [53], for both the IG and CG);
2. satisfaction with the intervention (for both the IG and CG);
3. changes in patients’ attitudes (for example concerning immunotherapy; for both the IG and CG);
4. structural barriers or barriers with regard to content, which hinder patients to actively participate in decision-making;
5. promoting factors; and
6. influence of coaching on patient-physician communication.

Questionnaires (with some open questions; for both the IG and CG), videos of consultations (IG), and interviews (for both the IG and CG) and/or focus groups (for both the IG and CG) will be used for the evaluation. Some aspects are already covered by primary and secondary endpoint questionnaires. Subgroup analyses are intended to determine whether coaching of patients seeking a change of immunotherapy has a greater or smaller effect compared to treatment-naïve patients.

### Maintenance

The collection of possible behavioural changes in decision coaches can provide important information to explore which factors serve to maintain the implementation of the intervention or have a limiting influence. The following aspects will be covered for decision coaches:

1. DECIMS-Wiki-use as a potential factor,
2. change of DECIMS-Wiki use in the course of the study,
3. self-assessed changes in knowledge and skills (for example coaching skills) during the study,
4. use of the materials (moderation cards and patient workbook),
5. willingness to work and train in the new field of activity,
6. self-assessed change in attitude of nurses in the course of the study (for example, in terms of coaching and about immunotherapies (see also nurses and response of cluster)).

The following aspects will be covered for patients:

1. factors that lead to reconsidering the decision for or against immunotherapy (for both the IG and CG),
2. DECIMS-Wiki use as a potential factor (for both the IG and CG), and
3. contact with the decision coach after the coaching session(s) (IG).

### Unintended consequences

#### Patients

Potentially, negative as well as positive effects may be caused by the intervention. Therefore, security parameters (HADS [61] and HAQUAMS [62]) are applied to assess positive and negative changes in patients. In addition, other possible effects of the intervention will be identified on the basis of interviews and questionnaires.

#### Decision coaches

It will be assessed (by questionnaires and interviews) whether the training or coaching evokes unintended consequences such as anxiety, burden within the situation, and/or a conflict between their beliefs or current practice in the outpatient clinics and the content of the intervention, in trained nurses.

#### Physicians (intervention group)

Possible effects of the intervention on the relationship between physicians and patients and physicians and trained nurses will be evaluated via questionnaires and interviews.

### Theory

The Theory of Planned Behaviour (TPB) is based on the assumption that behaviour is largely the result of setting, beliefs, and expectations regarding future events. When weighing different alternatives, an individual will choose the action that most likely causes a positive result. According to the theory of planned behaviour, the domains ‘attitude’, ‘subjective norm’, and ‘perceived behavioural control’ determine the behaviour of a person. In a previous project, a questionnaire based on the theory has been developed in order to elaborate the intended behaviour respective to a decision of patients with MS on immunotherapy [57]. This is one of the questionnaires used in the trial.

Beyond that, the development of the training programme for nurses was guided by the theory of planned behaviour, and the theory will be considered and used in the development of the process evaluation questionnaires to identify barriers and supporting factors. Beyond the TPB as on underlying framework of this project, the concepts of SDM, evidence-based medicine, and EBPI have contributed significantly to the development and the contents of the intervention [46,75].

### Data analysis (process evaluation)

As described by Creswell and Plano Clark [78], the main steps for the data analysis in the embedded mixed-methods design are:

1. analysis of the primary data set (trial data, see Table 1),
2. analysis of the secondary data (process data),
3. specification of dimensions by which the results should be compared,
4. specification of what information from dimensions should be compared,
5. comparison of data sources, and
6. data interpretation according to the research questions (in which way do secondary data sets contradict, augment, or support trial results?).

First, the process evaluation and trial data will be analysed separately. After that, the data will be connected and the results will determine the interview questions. Finally, all data sets will be merged (joint display).

The trial endpoint data analysis will be performed according to the protocol. Quantitative process evaluation data (surveys and evaluation forms) will be analysed descriptively using SPSS (International Business Machines Corporation (IBM), Armonk, United States of America) or R (R Development Core Team) software. Some subgroup analyses will be performed (for example, regarding the start or change of immunotherapy and decision type) in order to explore the impact of the intervention on different groups. Interviews will be analysed by content analysis [81] and coded thematically with a specific software programme (QCAmap (P. Mayring and T. Fenzl), Klagenfurt, Germany)

Qualitative data analysis will be guided by the TPB.

### Summary process evaluation

The framework of Grant *et al*. [70] facilitates systematically retrieving, appraising, and analysing important aspects of the complex intervention of decision coaching. The planned questionnaires allow for an elaborate interpretation of study results. In addition, the qualitative interviews enable further exploration of facilitators and barriers concerning the implementation of the intervention in different centres with different structures and processes, as well as different groups of people. The process evaluation offers the opportunity to capture the way in which the complex intervention causes effects, and to determine factors that have a supporting or hindering influence. Intentionally, besides some open questions in the evaluation forms, no qualitative data is collected during the trial, so as not to interfere with the processes of the complex intervention. However, important potential problems can be detected by regular telephone calls with the nurses of all centres. Through qualitative interviews and possibly focus groups after the trial, it is possible to further elaborate on the results of the quantitative questionnaires. Due to the interpretation of the data, new questions may be raised that can be addressed in the interviews. All quantitative questionnaires of the process evaluation were specified and created before the beginning of the trial. The qualitative interview guides are created after the completion of the study, in order to respond with flexibility, for example to unexpected events.

## Discussion

The proposed cluster randomised controlled trial aims to assess the effectiveness of a new model of patient decision support concerning MS immunotherapy options in Germany. As this intervention is associated with substantial structural changes, as for example nurses in Germany seldom explain treatment options, the trial is accompanied by a thoroughly developed mixed-methods research process evaluation in order to explore the underlying processes.

This is the first cluster randomised controlled trial where a nurse-led immunotherapy decision coaching intervention in persons with RRMS is evaluated. This study responds to Stacey *et al*.’s [16] call for more research to evaluate the value of decision coaching beyond patient decision aids.

In conclusion, this trial will investigate whether patients with MS who are facing an immunotherapy treatment decision will benefit from decision coaching delivered by trained nurses.

## Trial status

Patient recruitment for the trial started in autumn 2014.

## Additional files

Additional file 1:
**DECIMS SPIRIT 2013 Checklist [** 82 **-** 90 **].**
Additional file 2:
**Lead investigators in participating centres and ethical committees.**
Additional file 3:
**Overview process evaluation.** CRCT: Cluster randomised controlled trial; EBM: Evidence-based medicine, EBPI: Evidence based patient information, SDM: Shared decision making. Additional file 4:
**Instruments DECIMS process evaluation.** EF: evaluation form; IG: intervention group; CG: control group.

## Abbreviations

CG: Control group
CPS: Control Preference Scale
CSES: Coping self-efficacy scale
DCS: Decisional Conflict Scale
DGN: *Deutsche Gesellschaft für Neurologie* (German Society of Neurology)
DSMB: Data and Safety Monitoring Board
DMSG: *Deutsche Multiple Sklerose Gesellschaft* (German MS Self-help Society)
EBPI: Evidence-based patient information
EDSS: Expanded Disability Status Scale
HADS: Hospital Anxiety and Depression Scale
HAQUAMS: Hamburg Quality of Life in MS Scale
HCR trust scale: Health care relationship trust scale
IG: Intervention group
KKNMS: *Krankheitsbezogenes Kompetenznetz Multiple Sklerose* (Competence Network Multiple Sclerosis)
MAPPIN’SDM: Multifocal Approach to Sharing in Shared Decision Making
MMIC: Multi-dimensional measure of informed choice
MMR: Mixed methods research
MS: Multiple sclerosis
MRC: Medical Research Council, MRI, Magnetic resonance imaging
PBMS: Planned Behaviour in MS Scale
RIMS: Rehabilitation in Multiple Sclerosis
RRMS: Relapsing-remitting multiple sclerosis
SDM: Shared decision-making
SDMT: Symbol Digital Modalities Test
