# Supplementary material for: Evaluator-blinded trial evaluating nurse-led immunotherapy DEcision Coaching In persons with relapsing-remitting Multiple Sclerosis (DECIMS) and accompanying process evaluation: study protocol for a cluster randomised controlled trial
Source: Trials. 2015 Mar 21;16:106. doi: 10.1186/s13063-015-0611-7 (PMC4397890; doi:10.1186/s13063-015-0611-7)
Supplement: Additional file 1: — DECIMS SPIRIT 2013 Checklist [ 82 - 90 ]. [file 13063_2015_611_MOESM1_ESM.docx]

**Additional file 1: DECIMS - SPIRIT 2013 Checklist**

| **Section** | **Item Number** | **Included in publication (Y (yes) or described below** |
| --- | --- | --- |
| **Administrative Information** |  |  |
| Title | 1 | Y |
| Trial registration | 2a | Y |
|  | 2b | Y |
| Protocol version | 3 | 13.02.2015, Version 8a |
| Funding | 4 | Y |
| Roles and responsibilities | 5a | Y  Christoph Heesen and the research team will be responsible for the following points:   - design of DECIMS - conduct of DECIMS - preparation of study protocol and revisions - preparation of case report files (CRFs) - organising steering committee meetings - analysis of study results - publication of study results.   Data collection and completion of CRFs as well as follow up of study patients will be monitored by AR. |
|  | 5b | **Bundesministerium für Bildung und Forschung [Federal Ministry of Education and Research]**  Projektträger im Deutschen Zentrum für Luft- und Raumfahrt e.V.  Gesundheitsforschung  Dr. Svenja Diekhoff  Heinrich-Konen-Str. 1  D-53227 Bonn  **Krankheitsbezogenes Kompetenznetz Multiple Sklerose [Competence Network Multiple Sclerosis]**  Klinikum rechts der Isar  Prof. Dr. B. Hemmer  TU München  Neurologische Klinik und Poliklinik  Ismaninger Straße 22  D-81675 München |
|  | 5c | Y |
|  | 5d | **Steering committee:**  F. Paul (Berlin), R. Diem (Heidelberg), I. Kleiter (Bochum), CH and SK.  All steering committee members have to agree to the final protocol.  Further responsibilities: Reviewing progress of study and if necessary agreeing changes to the protocol to facilitate the conduction of the study.  **Data Manager**  G. Antony  G. Antony is responsible for the maintenance of the trial IT system and data verification.  **Data and safety monitoring board (DSMB):**  A. Solari (Milano), G. Giovannoni (London), D. Stacey (Ottawa), H. Leino-Kilpi (Helsinki)  Principal Investigator (CH) and research team (AR, SK, JK, EV) |
| **Introduction** |  |  |
| Background and rationale | 6a | Y |
|  | 6b | Y |
| Objectives | 7 | Y |
| Trial design | 8 | Y |
| **Methods** |  |  |
| Participants, interventions,  and outcomes | | |
| Study setting | 9 | Y |
| Eligibility criteria | 10 | Y |
| Interventions* | 11a | Y |
|  | 11b | Y |
|  | 11c | Y |
|  | 11d | Y |
| Outcomes | 12 | Y |
| Participant timeline | 13 | Y |
| Sample size | 14 | Y |
| Recruitment | 15 | Y |
| Assignment of interventions |  |  |
| Allocation |  |  |
| Sequence generation | 16a | Y |
| Allocation concealment mechanism | 16b | Y |
| Implementation | 16c | Y |
| Blinding (masking) | 17a | Y |
|  | 17b | Y |
| Data collection,  Management and analysis | | |
| Data collection methods | 18a | **Primary endpoint**  Informed choice (MMIC [82]) including the sub-dimensions risk knowledge, attitude and uptake is the primary endpoint.  A modified version of the questionnaire has been tested ahead of the main trial through a web-based survey in order to evaluate acceptability and design changes. The questionnaire has shown robust results in a cohort of n=705 MS patients with a mean of 9,58 correct items out of 19 items (SD 3.28), showing normal distribution and good internal consistency (Cronbach's alpha, 0,8).  **Secondary endpoints**   1. Decisional Conflict Scale (DCS): Decisional Conflict [54] is the key secondary endpoint. The English language scale version was evaluated in 909 individuals and had a test-retest reliability coefficient of 0.81 [83]. The German translation has been conducted by Buchholz and colleagues [84]. However, we will apply the dyadic version of the scale [54] which has been recently validated in a large MS cohort within the PERCEPT study [85] measuring patients’ and nurses’ as well physicians’ perspective. 2. Control Preference Scale (CPS): Autonomy preference will be assessed using a web-based card set (CPS [56]), which has recently been validated, showing satisfactory results concerning reliability [86]. 3. Planned Behaviour in MS (PBMS): Decision making processes concerning immunotherapy will be assessed using the PBMS, which has been developed by our research group in German language [57]. The questionnaire showed sensitivity to change in a recent RCT [38]. 4. The Coping-Self-Efficacy-Scale has been developed in the context of HIV behavioural interventions [58]. It integrates a coping instrument and a self-efficacy measure asking patients for their confidence in applying the right coping strategy depending on a given challenge. The German version has been applied in a cross-sectional study [87] and has been used in pilot work on a behavioural intervention in MS showing validity of the tool (unpublished data). The longer questionnaire CSES version has been also applied in MS patients [88]. 5. MAPPIN’SDM as an additional measure of SDM based on fitting of physicians’ and nurses’ perceptions with patients views [59]. All coaching sessions will be videotaped and at least a randomised cohort of eight coaching’s from each centre will be rated independently by two researches with the observer-based instrument. 6. The HCR trust scale has been developed by Bova et al [89] and has been translated from English to German for the current study. The English version produced acceptable reliability by illness status and gender as well as in multiple age groups [89].     **Assessment of safety**   1. Emotional distress will be measured using the Hospital Anxiety and Depression Scale (HADS), which has been used in different MS studies [38, 39]. 2. Disease specific quality of life will be evaluated using the Hamburg Questionnaire on Quality of Life in MS, which has shown validity and reliability (HAQUAMS [62] updated in [90]). 3. Disability: Expanded-Disability-Status-Scale (EDSS [63]) and perceived progression (HAQUAMS). 4. Cognition: Symbol-Digit-Modalities-Test (SDMT) measuring information processing as a widely accepted screening tool for cognitive dysfunction in MS [64]. Together, these two measurements (EDDS and SDMT) are suitable to describe and compare the baseline characteristics of patients concerning MS disease status.   Data collection forms in German language are available on request. |
|  | 18b | **Retention**  As successfully performed in previous studies, we aim to promote retention of study patients using email and telephone reminders. Patients will be asked to complete questionnaires within a pre-specified time period. Study participants who miss the proposed completion period will be reminded per email (after 7 and 14 days). Individuals, who still have not filled in the questionnaires after three weeks, will be contacted by telephone. Information about expected time to fill in the questionnaires will be provided by reminder emails (see also strategies to improve adherence). |
| Data management | 19 | All electronic data will be captured and processed through the IT platform of the KKNMS supervised by G. Antony who will be unaware of patients’ allocation. Patients will receive an email with the username as well as a second email with a database link and need to generate a password to gain access to the questionnaires. Therefore, data entry by the research team will only be necessary on special occasions, e.g. when patients feel unable to complete the web-based questionnaires. In those cases, data will be entered by blinded members of the coordinating centre in Hamburg. All data will be entered by one team member. Nevertheless, to ensure data accuracy, entered data will be controlled by two blinded members of the research team in Hamburg at the end of the study. Data will be pseudonymised with clusters coded using numbers and patients using a mixed code with letters and numbers, the latter will be automatically generated by the database. Electronic and paper based data will be stored for 10 years at a save place at the University of Hamburg. |
| Statistical methods | 20a | Y |
|  | 20b | Y |
|  | 20c | Y |
| Monitoring |  |  |
| Data monitoring | 21a | A data and safety monitoring Board (DSMB) will be established with international experts who are not involved in the current study. The DSMB will receive annually reports concerning adherence to the study protocol and standards of good clinical practice. |
|  | 21b | As relevant adverse events are unlikely, no interim analyses are planned, no stopping rules applied. However, the DSMB can demand the conduction of an interim analysis and subsequently give advice whether to continue, modify, or stop the trial, and provide the funding organisation with information and advice. The DSMB will be independent from the study sponsor. |
| Harms | 22 | Y |
| Auditing | 23 | There are no planned audits. However, regulatory authorities might choose to audit the study. |
| **Ethics and dissemination** |  |  |
| Research ethics approval | 24 | Y |
| Protocol amendments | 25 | The DSMB will need to approve major changes of the study protocol e.g. concerning outcome measures. Also an amendment of the study protocol would be submitted to the ethical committees. Minor changes to the protocol, which will not affect the conduction of the study, will be communicated to the DSMB. Information about changes will be added to the study registration. |
| Consent or assent | 26a | Informed consent will be obtained by a person in the participating centres involved in the study i.e. a physician or a (study) nurse. |
|  | 26b | Not applicable |
| Confidentiality | 27 | All personal information will be entered into the database by participants or nurses (at baseline and logbook) and in exceptional cases by study managers. Data will be pseudonymised and there will be no possibility to link data to persons without access to the code list. Through the personal access of patients to the DECIMS-Wiki based on a personal account (sent via email), it will be possible to individually track the use of the DECIMS-Wiki (e.g. frequency, use of different parts), which will be used to analyse the DECIMS-Wiki use as well as the value of different parts of the DECIMS-Wiki. This will be performed with ExtraWatch, an encapsulated plugin to the Joomla platform. Therefore, the DECIMS-Wiki users IP address will be stored by the system but shielded to anyone but the system administration by G. Antony, C. Heesen and A. Rahn. However, data analyses of DECIMS-Wiki use will be based on pseudonyms. |
| Declaration of interests | 28 | Y |
| Access to data | 29 | The study centre will coordinate the intra-study data sharing process. All principal investigators will be given access to the cleaned data sets. |
| Ancillary and post-trial care | 30 | Not applicable. |
| Dissemination policy | 31a | Results will be published in major journals and presented at scientific conferences as e.g. European Committee for Treatment and Research in Multiple Sclerosis (ECTRIMS), Rehabilitation in Multiple Sclerosis (RIMS), International Shared Decision Making Conference (ISDM), European Association for Communication in Healthcare (EACH). Furthermore, it is planned to publish main results on relevant patient websites. All patients will receive structured feedback and copies of major publications.  If the trial is successful, an implementation study will be applied for to transfer the findings into care. |
|  | 31b | Authorship will be shared between persons involved in the study following the current guidelines of the International Committee of Medical Journal Editors (ICMJE). No professional writers will be employed and no persons not directly involved in the study will be granted authorship. |
|  | 31c | Y  It is not planned to make the data set and statistical code publicly accessible, but on request from researchers, individual data will be provided. |
| **Appendices** |  |  |
| Informed consent materials | 32 | Available on request in German language |
| Biological specimens | 33 | Not applicable |
