# Supplementary material for: Evaluator-blinded trial evaluating nurse-led immunotherapy DEcision Coaching In persons with relapsing-remitting Multiple Sclerosis (DECIMS) and accompanying process evaluation: study protocol for a cluster randomised controlled trial
Source: Trials. 2015 Mar 21;16:106. doi: 10.1186/s13063-015-0611-7 (PMC4397890; doi:10.1186/s13063-015-0611-7)
Supplement: Additional file 2: — Lead investigators in participating centres and ethical committees. [file 13063_2015_611_MOESM2_ESM.docx]

**Additional file 2: Lead investigators in participating centres and ethical committees**

| **Lead investigators in participating centres** |  | **Ethical committee** |
| --- | --- | --- |
| **Orhan Aktas, Prof. Dr.**  Neurologische Klinik  Heinrich-Heine-Universität Düsseldorf |  | Ethikkommission der Medizinischen Fakultät der Heinrich-Heine-Universität Düsseldorf (reference no.: 4681) |
| **Martin Berghoff, Dr.**  Klinik und Poliklinik für Neurologie  Universitätsklinikum Gießen |  | Ethik-Kommission am Fachbereich Medizin (Justus-Liebig Universität Giessen) (reference no.:82/14) |
| **Ricarda Diem, Prof. Dr.**  Neurologische Klinik  Universität Heidelberg |  | Ethikkommission der Med. Fakultät Heidelberg (reference no.: S-264/2014) |
| **Jürgen H. Faiss, Prof. Dr.**  Klinik für Neurologie und Neurophysiologie  Asklepios Fachklinikum Teupitz |  | Ethik-Kommission der Landesärztekammer Brandenburg (reference no.: AS 92(bB)/2014) |
| **Christoph Heesen (CH), Prof. Dr.**  Institut für Neuroimmunologie und klinische Multiple Sklerose Forschung (inims)  Universitätsklinikum Hamburg-Eppendorf |  | Ethik-Kommission der Ärztekammer Hamburg (reference no.: PV4576) |
| 1. **Frank A. Hoffmann, Dr.** 2. Klinik für Neurologie 3. Krankenhaus Martha-Maria Halle-Dölau |  | Ethical committee: Ethikkommission der Ärztekammer Sachsen-Anhalt (reference no.: 42/14) |
| **Ingo Kleiter, Prof. Dr.**  Neurologische Klinik  St. Josef-Hospital Bochum |  | Ethik-Kommission der Med. Fakultät der Ruhr Universität Bochum (reference no.: 4846-13) |
| **Luisa Klotz, Dr.**  Klinik für Neurologie  Universitätsklinikum-Münster |  | Ethik-Kommission der Ärztekammer Westfalen-Lippe und der Medizinischen Fakultät der Westfälischen Wilhelms-Universität (reference no.: 2014-235-b-S) |
| **Wolfgang Köhler, Dr.**  Klinik für Neurologie und neurologische Intensivmedizin  Fachkrankenhaus Hubertusburg, Wermsdorf |  | Ethikkommission bei der Sächsischen Landesärztekammer (reference no.: EK-BR-80/14-1) |
| **Mathias Mäurer, Prof. Dr.**  Klinik für Neurologie  Caritas Krankenhaus Bad Mergentheim |  | Ethik-Kommission der Friedrich-Alexander Universität Erlangen Nürnberg (reference no.: 191_14 Bc) |
| **Friedemann Paul, Prof. Dr.**  NeuroCure Clinical Research Center  Charité – Universitätsmedizin Berlin, Campus Mitte |  | Ethikausschuss 1 am Campus Charité - Mitte (reference no.: EA1/151/14) |
| **Alexander Simonow**  Neurologische Klinik Sorpesee |  | Ethik-Kommission der Ärztekammer Westfalen-Lippe und der Medizinischen Fakultät der Westfälischen Wilhelms-Universität (reference no.: 2014-235-b-S) |
| **Susanne Windhagen, Dr.**  MVZ Multiple Sklerose  Klinikum Osnabrück |  | Ethikkommission der Ärztekammer  Niedersachsen (reference no.: Grae/109/2014) |
| **Uwe Zettl, Prof. Dr.**  Klinik und Poliklinik für Neurologie  Universitätsmedizin Rostock |  | Ethikkommission an der Medizinischen Fakultät der Universität Rostock (reference no.: A 2014-0073) |
