# Supplementary material for: Evaluator-blinded trial evaluating nurse-led immunotherapy DEcision Coaching In persons with relapsing-remitting Multiple Sclerosis (DECIMS) and accompanying process evaluation: study protocol for a cluster randomised controlled trial
Source: Trials. 2015 Mar 21;16:106. doi: 10.1186/s13063-015-0611-7 (PMC4397890; doi:10.1186/s13063-015-0611-7)
Supplement: Additional file 3: — Overview process evaluation. CRCT: Cluster randomised controlled trial; EBM: Evidence-based medicine, EBPI: Evidence based patient information, SDM: Shared decision making. [file 13063_2015_611_MOESM3_ESM.doc]

**Additional file 3:** Overview process evaluation

| **Overview process evaluation DECIMS** | | | |
| --- | --- | --- | --- |
| **Domain** | **Objects of investigation** | **Ascertainment** | **Time point** |
| **Context** | Context factors in Germany (health system) | Description | Pre-intervention |
|  | Barriers and promoting factors in the participating outpatient clinics | Visits in the participating centres, survey | Pre-intervention |
| **Recruitment of clusters** | Cluster recruitment  Reason for study participation  Cluster-specific differences | Documentation of cluster recruitment  Survey (physicians and nurses)  Interviews, surveys | Pre-, during and post-intervention |
| **Delivery to clusters**  **Staff level** | Development of the intervention | Visits in the participating centres, piloting of study materials (e.g. think aloud), feasibility and pilot study | Pre-intervention |
|  | Delivery of the intervention to nurses (participation, reach, attitude) | Evaluation forms, documentation, knowledge questionnaire | Pre-intervention |
|  | Delivery of the recruitment strategy to all centres | Documentation of participation in web-conferences | Pre-intervention |
| **Response of clusters** | Stakeholders (intervention and control group): attitude in participating centres (lead investigator, physicians, nurses) respective the intervention | Evaluation forms, interviews | Pre- and post-intervention |
|  | Decision coaches (trained nurses): coaching performance (delivered as intended)  E.g.: acquired routine, barriers attitude and willingness to work and further train in the new action field | Evaluation form, video recording (coaching), logbook, interviews | Pre-, during and post-intervention |
|  | Physicians (intervention and control group): change in routine through the intervention | Evaluation form, interviews | Pre- and post-intervention |
|  | Nurses  (control group): change in routine through the control intervention | Evaluation form, interviews | Pre- and post-intervention |
| **Recruitment & reach in individuals** | Non-responder analysis | Checklist | During the intervention |
|  | Recruitment procedure | Web-based call, documentation of study, recruitment (screening lists) | Pre- and during intervention |
| **Delivery to individuals**  **(Dose delivered)** | Intervention group: delivery of the intervention to individuals (decision coaching and DECIMS-Wiki) | Video recording (coaching), evaluation form, interviews | During and post-intervention |
|  | Control group: delivery of the control intervention to individuals (DECIMS-Wiki) | Evaluation form, interviews | Post-intervention |
| **Response of individuals**  **(Dose received)** | E.g.: Satisfaction with the intervention, knowledge, attitude, barriers and facilitators | Questionnaires (primary and secondary endpoints CRCT), evaluation form, interviews | Post-intervention |
| **Maintenance** | Decision coaches: Knowledge and attitude, acquired routine, coaching performance DECIMS-Wiki use | Questionnaire, evaluation form, video recording (coaching), interviews | Pre- during and post-intervention |
|  | Patients: further needs (coaching, DECIMS-Wiki), autonomy preferences, knowledge | Evaluation form, questionnaires (primary and secondary endpoints CRCT), interviews | During and post-intervention |
| **Unintended consequences** | Decision coaches: Stress, professional relationship to physicians and patients, barriers | Evaluation form, video recording (coaching), interviews | During and post-intervention |
|  | Patients: anxiety, barriers, physician contact, negative impact on quality of life | Evaluation form, questionnaires (security parameters CRCT), Interviews | During and post-intervention |
|  | Physicians: professional relationship to nurses and patients, barriers | Evaluation form, interviews | During and post- intervention |
| **Theory** | TPB, SDM, EBPI, EBM | Application during study planning and the development of study materials, used in evaluation forms, during video analysis | Pre- during and post-intervention |
