# Supplementary material for: Evaluator-blinded trial evaluating nurse-led immunotherapy DEcision Coaching In persons with relapsing-remitting Multiple Sclerosis (DECIMS) and accompanying process evaluation: study protocol for a cluster randomised controlled trial
Source: Trials. 2015 Mar 21;16:106. doi: 10.1186/s13063-015-0611-7 (PMC4397890; doi:10.1186/s13063-015-0611-7)
Supplement: Additional file 4: — Instruments DECIMS process evaluation. EF: evaluation form; IG: intervention group; CG: control group. [file 13063_2015_611_MOESM4_ESM.doc]

**Additional file 4: I**nstruments DECIMS process evaluation

| **Evaluation MS-outpatient clinics (nurses, physicians and decision coaches)** | | | | | | | | | |
| --- | --- | --- | --- | --- | --- | --- | --- | --- | --- |
| **Instrument/group** | **Pre centres randomisation** | | **Pre-training** | | **Post-training** | **Begin of study recruitment** | **6 months after study recruitment** | | **12 months after study recruitment** |
| **Survey outpatient clinics (lead investigators)** | **x** | |  | |  |  |  | |  |
| **EF (IG): Decision-Coaches** |  | | **x** | | **x** | **x** | **x** | | **x** |
| **EF (IG): physicians** |  | |  | |  | **x** |  | | **x** |
| **EF (IG): untrained nurses** |  | |  | |  | **x** |  | | **x** |
| **EB (CG): nurses** |  | |  | |  | **x** |  | | **x** |
| **EF (CG): physicians** |  | |  | |  | **x** |  | | **x** |
| **EB (CG): untrained nurses** |  | |  | |  | **x** |  | | **x** |
| **Video records (decision coaches)** |  | |  | | **2 patients (run-in)** |  |  | |  |
| **Structured telephone calls IG: decision coach** | **Monthly during the first three months and every two to three months afterwards during study recruitment** | | | | | | | | |
| **Structured telephone calls CG: nurse** | **Monthly during the first three months and every two to three months afterwards during study recruitment** | | | | | | | | |
| **Interviews IG: decision coaches** | **After the study is finished** | | | | | | | | |
| **Interviews IG and CG: some nurses and physicians** | | **After the study is finished** | | | | | | | |
| **Evaluation patients (IG and CG)** | | | | | | | | | |
| **Instrument/group** | **Baseline** | | | **Directly after final physician decision encounter** | | **3 months after final physician encounter** | | **6 months after final physician encounter** | |
| **EF patients IG** | **x** | | | **x** | | **x** | | **x** | |
| **EF patients CG** | **x** | | | **x** | | **x** | | **x** | |
| **Video records decision coaching** | **All coaching sessions (every patient)** | | | | | | | | |
| **Logbook IG (filled in by decision coaches)** | **Baseline after every coaching and until the final physician decision encounter** | | | | | | | | |
| **Logbook CG (filled in by nurses)** | **Baseline and until the final physician decision encounter** | | | | | | | | |
| **Screening forms (filled in by physicians)** | **Has to filled in by physicians for every patient** | | | | | | | | |
| **Interviews patients (IG and CG)** | **Purposeful sampling after the study is finished** | | | | | | | | |
